# Annotations

**Published:** 1904-09-10

**Authors:** 


### Annotations.

### Motor-cars and Dust.

The advantagees and attractions of the motor-car, at least to those who do not boast the possession of such a vehicle, are largely qualified by several obvious inconveniences. In addition to the nerve-startling horn, the odour of petroleum which poisons the air, and the risk of a sudden and violent death, the car in dry weather raises a cloud of dust which afflicts not only pedestrians and other travellers, but penetrates into the houses situated near the road-side. Not unnaturally there are many complaints and denunciations, and threats are issued against the liberty of those who use the new mode of locomotion. But if the grumblers are to obtain relief they must order their policy wisely. It must be taken for granted that the motor-car has come to stay. The fashion of the hour may, it is true, change, but the obvious utility of the car will ensure for it a permanent and ever-extending employment. Hence the right method of procedure is not to denounce the use of the motor—for this indeed is a vain hope—but to advocate the establishment of conditions under which it can be used without becoming a public nuisance and danger. For those who suffer from the dust it creates we have a very real sympathy, and it may be that their grievance is even more serious than they know. The mechanical properties of dust are bad enough in all conscience, but its bacteriological possibilities may be much worse. Those who have charge of large out-patient departments are well aware of the difference produced by a very wet summer, such, for example, as that of 1903, in the number of patients suffering from various forms of sore throat, diarrhœa, etc. This is a very suggestive fact in relation to the influence of dust on disease, and the motor-car by stirring up the subject, in a literal as well as in a figurative sense, may be a blessing in disguise. To propose to abolish the car is mere wild talk to which no reasonable person will listen. But a proposal that local authorities shall be compelled to construct roads of suitable material is quite within the range of practical politics. And if sufferers from the dust caused by motor-cars can effect this reform, they will do much more than [protect themselves from inconvenience. An agitation which springs from personal interest has before now proved a lever in the promotion of the public welfare.

### A Glasgow Housing Experiment.

Professor Wm. Smart, LL.D., a leading member of the commission appointed to consider the question of housing in Glasgow, has given some of the reasons which decided the commission to advise a limited scheme of municipal building and owning. Admitting the force of the argument against the general policy Professor Smart has pointed out that if the laws against overcrowding and other insanitary conditions were sternly put in force in Glasgow, some 15,000 to 20,000 persons would be dispossessed, and would not be able to find houses at the rents they had previously been paying. Further, this inability would be largely due to municipal requirements, such as the provision of 400 feet of air space for each adult, ample sanitary appliances, and a certain thickness of walls and solidity of construction—the latter, in the opinion of many builders, quite in excess of the necessities of the position. To meet all this the commission has advised the municipality to erect a limited number of tenements of one or two apartment houses for respectable people of the poorest class, such houses to be under the charge of efficient caretakers, and to be erected near to the area of dispossession. In addition, the commission advises that some provision be made for a certain type of the "non-respectable" persons to be evacuated from the condemned areas. The criminal and hopelessly dissolute are without claim on the community, but there are numerous persons who, though without character, are not in these groups, and it were well to meet these by the provision of plain, easily cleansed houses, tenancy of which was under strict regulations. This would afford a chance to these people to re-establish their characters, and to prove themselves worthy of a better class of dwelling. The scheme is admittedly an experiment, but it is an experiment which will command much sympathy, and will be watched with great interest. Perhaps the strongest claim in its favour is that it removes the last excuse for not going systematically and vigorously forward in the policy of demolition of insanitary houses and prevention of overcrowding.

### A Twentieth-Century Sanitarian.

While it is admitted that the housing question is not yet by any means solved in our large towns, most of us thought that considerable improvements in our dwellings had been made within the last generation. Among these we have regarded the placing of properly fitted bathrooms in even modest dwellings. The bathroom, with its hot and cold water supply, is nowhere more valuable than in the homes of the respectable class, artisan or professional, whose means are small, who keep no servants, but who value personal cleanliness. But now arises one, who, declaring in the *Daily Chronicle* that the housing question is "certainly a terrible one from several points of view," offers as his solution the omission of the bathroom from six-roomed houses. "In most cases," he says, "the bath is too small for adults, and the room is used to put rubbish in." With regard to the latter point, we may assume that our would-be reformer knows the manners and customs of his own family and their friends, but he need not on that account malign all the other inhabitants of six-roomed houses. Indeed, it is just in small houses that a bathroom is most valuable. In large dwellings, where a sufficient number of servants are kept, it is simple enough for the members of the family to have their baths in their rooms. Though even there, if there is not a fitted bathroom provided for the servants, there is a risk that these will rather go unwashed than trouble to carry baths and hot water upstairs for themselves. But where only one servant, or none, is kept, the bath which can be got ready by simply turning on a tap is a great boon. If it is not as fully taken advantage of as might be, it is matter for regret, but certainly the provision of the bathroom has made cleanliness not only possible but popular among people who could not otherwise have achieved it, and it would indeed be a retrograde step to omit the provision of even a small bathroom in houses of moderate size and rent.

